# Physical Activity Program for the Survival of Elderly Patients With Lymphoma: Study Protocol for Randomized Phase 3 Trial

**DOI:** 10.2196/40969

**Published:** 2022-11-25

**Authors:** Jonas Dubu, Sébastien Boyas, Virginie Roland, Sébastien Landry, Anne-Lise Septans, Magali Balavoine, Hugues Bourgeois, Yoann Pointreau, Fabrice Denis, Christophe Letellier, Katell Le Dû

**Affiliations:** 1 ELSAN Institut inter-regional de Cancerologie Jean Bernard Le Mans France; 2 Motricite Interactions Performance (MIP) UR4334 Le Mans Universite Le Mans France; 3 WeproM Le Mans France; 4 Hematology Department, Centre Hospitalier de Perpignan Perpignan France; 5 Institut National de la e-Santé Le Mans France; 6 CNRS UMR 6614 - CORIA Rouen Normandie Universite Saint Etienne du Rouvray France; 7 Hematology Department, Confluent Private Hospital Nantes France

**Keywords:** diffuse large B-cell lymphoma, adapted physical activity, survival, sarcopenia

## Abstract

**Background:**

The practice of regular physical activity can reduce the incidence of certain cancers (colon, breast, and prostate) and improve overall survival after treatment by reducing fatigue and the risk of relapse. This impact on survival has only been demonstrated in active patients with lymphoma before and after treatment. As poor general health status reduces the chances of survival and these patients are most likely to also have sarcopenia, it is important to be able to improve their physical function through adapted physical activity (APA) as part of supportive care management. Unfortunately, APA is often saved for patients with advanced blood cancer. As a result, there is a lack of data regarding the impact of standardized regular practice of APA and concomitant chemotherapy as first-line treatment on lymphoma survival.

**Objective:**

This study aimed to assess the impact of a new and open rehabilitation program suitable for a frail population of patients treated for diffuse large B-cell lymphoma (DLBCL).

**Methods:**

PHARAOM (Physical Activity Program for the Survival of Elderly Patients with Lymphoma) is a phase 3 randomized (1:1) study focusing on a frail population of patients treated for DLBCL. The study will include 186 older adult patients with DLBCL (aged >65 years) receiving rituximab and chemotherapy. Overall, 50% (93/186) of patients (investigational group) will receive APA along with chemotherapy, and they will be supervised by a dedicated qualified kinesiologist. The APA program will include endurance and resistance training at moderate intensity 3 times a week during the 6 months of chemotherapy. The primary end point of this study will be event-free survival of the patients. The secondary end points will include the overall survival, progression-free survival, prevalence of sarcopenia and undernutrition, and patients’ quality of life. This study will be conducted in accordance with the principles of the Declaration of Helsinki.

**Results:**

Recruitment, enrollment, and data collection began in February 2021, and 4 participants have been enrolled in the study as of July 2022. Data analysis will begin after the completion of data collection. Future outcomes will be published in peer-reviewed health-related research journals and presented at national congress, and state professional meetings. This publication is based on protocol version 1.1, August 3, 2020.

**Conclusions:**

The PHARAOM study focuses on highlighting the benefits of APA intervention on survival during the period of first-line treatment of patients with DLBCL. This study could also contribute to our understanding of how an APA program can reduce complications such as sarcopenia in patients with lymphoma and improve their quality of life. By documenting the prevalence and relationship between sarcopenia and exercise load, we might be able to help physicians plan better interventions in the care of patients with DLBCL.

**Trial Registration:**

ClinicalTrials.gov NCT04670029; https://clinicaltrials.gov/ct2/show/NCT04670029

**International Registered Report Identifier (IRRID):**

DERR1-10.2196/40969

## Introduction

### Background

Diffuse large B-cell lymphoma (DLBCL) is the most common type of non-Hodgkin malignant lymphoma (representing 31% of lymphomas), with an incidence of 15 to 20 new cases per year per 100,000 inhabitants in France [1]. The median age of DLBCL diagnosis is 65 years, and one-third of the patients are aged >75 years [2]. Since the 2000s, the standard first-line treatment consists of 6 to 8 cycles of rituximab plus cyclophosphamide, hydroxydaunorubicin (doxorubicin), vincristine, and prednisone (R-CHOP) administration, and to date, no additional molecule has managed to demonstrate its superiority over the R-CHOP scheme in terms of the overall survival or event-free survival (EFS) [3,4]. The risk of relapse within 3 years after the first-line treatment in patients with DLBCL is 40%, and >50% of patients develop complications during the treatment [5]. Poor prognostic factors include age >60 years, high lactate dehydrogenase levels, an advanced stage of the disease, and an impaired general condition (as indicated by the National Comprehensive Cancer Network Prognostic Index) [6].

Several phase 3 trials have attempted to improve the survival of these patients by either offering maintenance treatment after an R-CHOP scheme with the use of an innovative molecule (such as enzastaurin in the Prevention of Relapse in Lymphoma Using Daily Enzastaurin trial [7]), everolimus in the Adjuvant everolimus in high-risk diffuse large B-cell lymphoma trial [8], and lenalidomide in the Study of Lenalidomide Maintenance Versus Placebo in Responding Elderly Patients With Diffuse Large B-Cell Lymphoma and Treated With Rituximab Plus Cyclophosphamide, Hydroxydaunorubicin (Doxorubicin), Vincristine, and Prednisone trial [3] or by combining an R-CHOP scheme with an experimental treatment, such as ibrutinib in the Randomized Phase III Trial of Ibrutinib and Rituximab Plus Cyclophosphamide, Doxorubicin, Vincristine, and Prednisone in Non–Germinal Center B-Cell Diffuse Large B-Cell Lymphoma trial [4] and venetoclax in the A Study Evaluating the Safety, Efficacy and Pharmacokinetics of Venetoclax Combined With Chemotherapy in Participants With B-Cell Non-Hodgkin’s Lymphoma and Diffuse Large B-Cell Lymphoma trial [9]. To date, only polatuzumab has demonstrated a significant improvement of progression-free survival (PFS) but not of overall survival in the Polatuzumab Vedotin in Previously Untreated Diffuse Large B-Cell Lymphoma trial [10].

One should not oversee the fact that other risk factors might be associated with relapse. Some of these factors are related to the lymphoma itself (coexpression of BCL2 or BCL6 and C-MYC markers, ABC profile, high metabolic tumor volume, etc) and others to the patient (comorbidities, vitamin D deficiency, hypoalbuminemia, etc) [6,11-16]. Nutritional disorders (obesity and undernutrition) and sarcopenia can also affect PFS and overall survival [17]. Sarcopenia is defined by a reduction in skeletal muscle mass and physical performance, that is, a decrease in muscular strength, overall physical activity, and walking, as well as the onset of balance disorders and falls [18-21], leading to (1) loss of muscle mass, (2) decreased strength, and (3) reduced physical performance [18]. Sarcopenia is diagnosed when at least criteria, (1) + (2) or (1) + (3) can be confirmed. The causes of sarcopenia are often multiple [19]: chronic diseases (including cancer), inflammatory diseases, endocrine dysfunctions, insulin resistance, undernutrition, sedentary lifestyle, aging, and certain anticancer treatments (chemotherapy, radiotherapy, targeted therapy, and corticosteroid administration) can lead to loss of muscle mass, muscle deconditioning, and even development of neuropathies and chronic fatigue [22].

The assessment of the severity of sarcopenia is based on anthropometric measurements (weight; height; skin folds; and waist, arm, and calf sizes in cm), quantification of muscular strength (handshake test and breathing test by peak flow meter or peak flow), and measurement of physical performance. Measurement of skeletal muscle mass by assessing the muscular surface at the L3 level of the spinal column through computed tomography (CT) is recommended [23,24]. Several studies have confirmed the negative effects of sarcopenia in patients with DLBCL. In a recent study of a cohort of patients (n=522) who received first-line treatment with R-CHOP, 47% of these patients were found to be sarcopenic and sarcopenia was significantly correlated with more hospitalizations owing to febrile neutropenia, higher treatment-related mortality, and a reduction in the dose intensity of chemotherapy [25]. Sarcopenia was also significantly associated with age >60 years and high comorbidity score [26]. Adverse effects and early discontinuation of R-CHOP treatment are known to have a negative impact on the overall survival and PFS of patients with DLBCL [26,27].

Treatment for sarcopenia can involve physical exercise and adequate protein intake [18]. Two studies have shown reduction in the risk of death when patients undertake physical activity with sufficient volume or intensity before starting their first-line treatment [28,29]. Interestingly, the benefit is maintained if the physical exercise is continued during and after chemotherapy [28]. A single study evaluated the impact of adapted and supervised physical activity on survival and response to treatment in patients with lymphoma (where several histological types were considered); the study revealed a significant increase in PFS of patients with lymphoma [30]. However, for the 42 patients with DLBCL in the same study, the results were inconclusive [30].

### Objectives

PHARAOM (Physical Activity Program for the Survival of Elderly Patients with Lymphoma) is the first randomized trial to evaluate the impact of a physical activity program on EFS of patients aged >65 years with previously untreated DLBCL. Sarcopenia will be screened in each group, and its impact on survival will also be assessed.

## Methods

### Study Design and Recruitment

**Figure 1:**
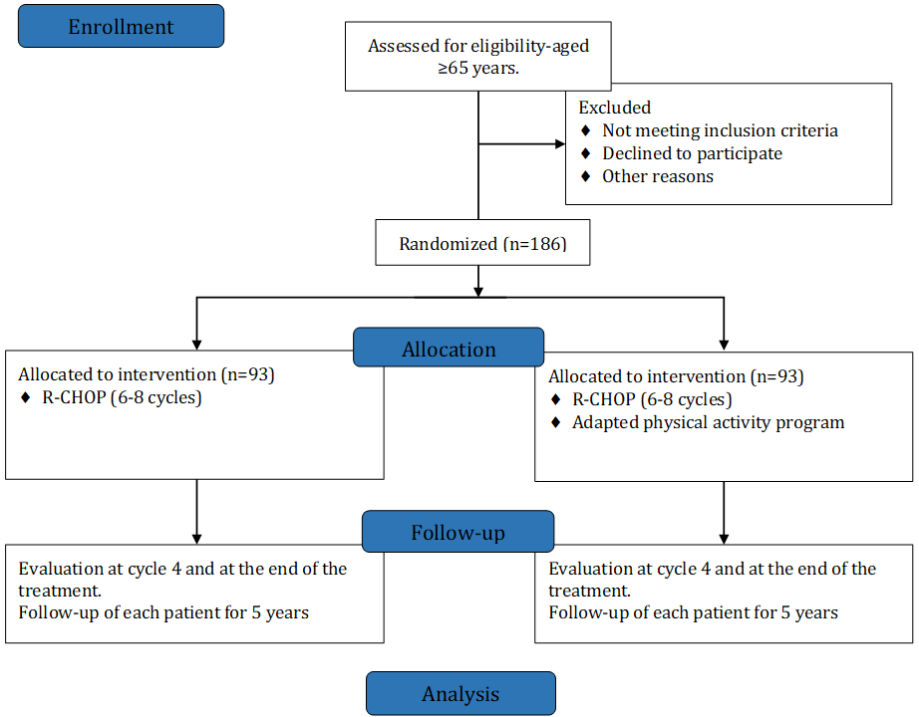
Flowchart of the PHARAOM (Physical Activity Program for the Survival of Elderly Patients With Lymphoma) study according to the usual CONSORT (Consolidated Standards of Reporting Trials) flowchart type. R-CHOP: rituximab plus cyclophosphamide, hydroxydaunorubicin (doxorubicin), vincristine, and prednisone.

This is a phase 3, open-label, randomized, multicenter, and prospective study. A total of 186 patients with DLBCL will be included: 93 (50%) will be randomized in the R-CHOP-alone arm and 93 (50%) will be included in the experimental arm (R-CHOP + physical activity program). A flowchart of the study is shown in Figure 1. We expected to detect an absolute difference of 15% in the EFS between the 2 groups. The final analysis will be performed at the end of the last patient’s follow-up, that is, at the 60th month after the last inclusion. Inclusions are planned for 36 months, and the follow-up period for each patient will have a duration of 60 months.

### Participants and Inclusion and Exclusion Criteria

The PHARAOM study will include patients who fulfill all the following criteria: patients with DLBCL regardless of their 2016 World Health Organization subtype classification [31] or a low-grade B lymphoma that has quickly transformed into a high-grade B lymphoma (follicular lymphoma of the marginal zone, mucosa-associated lymphoid tissue, and lymphocytic or lympho-plasma cells); patients aged >65 years; patients eligible for treatment with R-CHOP (left ventricular ejection fraction [LVEF] of >50%) regardless of the age-adjusted International Prognostic Index; patients with a performance status of <2 for treatment-naive patients or 2 cycles of chemotherapy (prephase or reduction phase treatment [Cyclophosphamide, Vincristine sulfate, Prednisone] and cycle #1 of R-CHOP) received owing to the development of a hemopathy; patients affiliated with a social security scheme; and patients providing written informed consent.

By contrast, patients with the following criteria will not be included in the study: patients with any other type of lymphoma (T lymphoma, Burkitt’s lymphoma, nontransformed low-grade B lymphoma, etc); patients with cerebral or meningeal damage related to hemopathy; deficit—acquired, congenital, motor, or sensory—that does not allow the implementation of adapted physical activity (APA) sessions; patients with uncontrolled arterial hypertension; patients with an LVEF of <50%; patients with a disabling heart or respiratory failure not allowing the completion of the APA sessions; patients with a disabling osteo-articular or muscular pathology; patients having received ≥3 cycles of the first-line chemotherapy; patients who are pregnant or breastfeeding; patients with an active infection of HIV, hepatitis C virus, or hepatitis B virus; patients lacking liberty (under guardianship or guardianship); patients with dementia, mental alteration, or a psychiatric pathology that may compromise the their ability to provide an informed consent, their compliance with the protocol, and the monitoring related to the trial; patients unable to perform the protocol follow-up for psychological, social, family, or geographic reasons.

### Sample Size

Given the experience gained by previous studies (particularly according to that of Feugier et al [32]), the sample size was calculated on the basis of the primary end point (EFS) to detect a difference of 15% at 24 months. Enrollment will take place for 36 months and follow-up for 60 months; thus, a significance level of 5% (2-sided) and power of 80% are required. Using the function plansurvct.func from the gsDesign (GNU General Public License) package of the R software and by planning an interim analysis at 50% of the event using DeMets–Lan and O’Brien–Fleming boundaries, we found it will be necessary to include 170 patients (85 per group) to obtain 114 events for the final analysis [33]. Moreover, considering that 10% of the patients could be lost during follow-up, we will need to enroll 93 patients per group (ie, 186 patients in total).

To be able to determine as quickly as possible whether the incorporation of APA into the follow-up of older adult patients with DLBCL improves EFS, we plan to perform an interim analysis that is scheduled to take place according to the DeMets–Lan limit at 50% of events [34]. To determine the number of patients needed to verify our hypothesis, we will ensure conforming to the following:



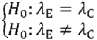



where λ_E_ and λ**_c_** are the hazard ratios for the APA (investigational) and usual care (control) groups, respectively. By setting the risk for wrongly rejecting the “null hypothesis” at 5% during the final analysis and the power at 80% (ie, 20% of risk for wrongly concluding that there is “no difference”), inclusions will be considered over 36 months. If H_0_ is not retained (*P*<.003), then we will conclude that APA produces a significant benefit for patients with DLBCL. In contrast, if H_0_ is not rejected (*P*>.003), then the study will be continued to provide us with all the required events necessary to obtain a definitive conclusion. The 1:1 randomization will be carried out following minimization with stratification on the center, age, sex, and comorbidities. The randomization will be performed by the investigator directly via the e-Case Report Form developed by the Ennov Clinical software.

### Primary and Secondary End Points

The primary objective of this study is to evaluate the effect of R-CHOP treatment combined with APA on EFS. EFS is defined as the time between the inclusion of patients and the date of the first event, such as relapse, infection, thrombosis, progression, or death, reported. The secondary objectives are to evaluate the compliance of the patients with the APA sessions and to quantify the overall physical activity load (in terms of volume and intensity) per patient and per session, overall survival, PFS, prevalence of complications (febrile neutropenia, anemia or thrombocytopenia requiring transfusion, infections, and venous or arterial thrombosis), prevalence of sarcopenia and nutritional disorders, patients’ quality of life, and cost of the required hospitalization.

### Survival

When the patient is censored, EFS is calculated as the time between the inclusion date and the date of the latest visit. The overall survival is defined as the time between the date of inclusion and the date of death (regardless of the reason for death) or the time between the date of inclusion and the date of the latest visit (if the patient is still alive). PFS is defined as the time between the date of inclusion and date of the first relapse, progression, or date of death (if no progression is seen before death), or date of the latest visit when the patient is censored.

### Evaluation Parameters

After inclusion, data will be collected from the medical records in the form of an e-Case Report Form (Ennov Clinical).

#### Nutritional Disorders

Screening for nutritional status disorders (overweight, obesity, undernutrition, and severe undernutrition) has been defined by the Haute Autorité de Santé [35,36]. Being overweight is defined as a BMI of >25 kg/m^2^ and obesity is defined as a BMI of >30 kg/m^2^. In contrast, malnutrition is defined according to the criteria presented in Textbox 1.

**Criteria for defining undernutrition and severe undernutrition in patients.**

**Undernutrition**

- Weight loss of >5% in 1 month or >10% in 6 months
- Albuminemia, albumin <35 g/L
- Mini Nutritional Assessment questionnaire global score <17

**Severe undernutrition**

- Weight loss of >10% in 1 month or >15% in 6 months
- BMI <18 kg/m^2^
- Albuminemia, albumin <30 g/L

The nutritional status (weight, height, BMI, albuminemia, and Mini Nutritional Assessment questionnaire) will be evaluated at the time of diagnosis, after 4 cycles of R-CHOP, and at the end of the treatment [37]. Moreover, the assessment of food intake (ingesta) using the visual analog scale is a good reflection of the average consumption of the patient. A threshold of <7 reflects a significant decrease in food intake that justifies a specialized treatment [37], and it will be offered during each cycle. This evaluation will be performed by the registered dietitian of each center or the investigating physician.

#### Sarcopenia

**Figure 2:**
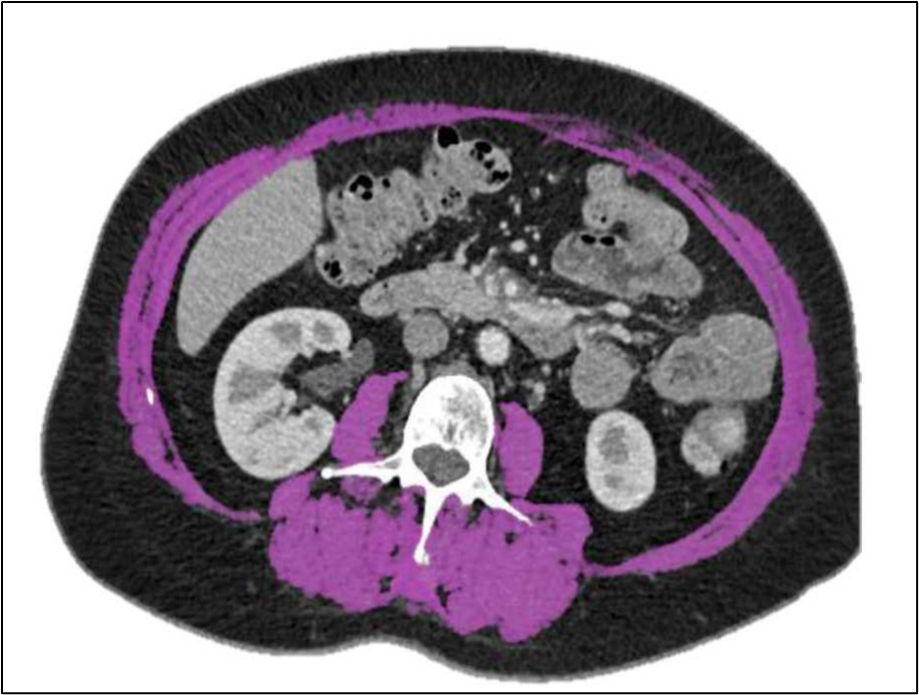
Illustration of the skeletal muscle segmentation (purple) at the third lumbar vertebra level (L3) using the ImageJ software in a 65-year-old female patient with diffuse large B-cell lymphoma.

The European Working Group on Sarcopenia in Older People and the Asian Working Group for Sarcopenia guidelines require the assessment of muscular strength, physical performance, and skeletal muscle mass for the diagnosis of sarcopenia. The third lumbar vertebra (L3) has been selected as the standard marker for the quantification of skeletal muscles using CT scans. The L3 muscle area is strongly associated with whole-body skeletal muscle mass (psoas, paravertebral, and abdominal wall muscles) [17]. The muscle area is measured on CT images [38] using the first automatic segmentation process of the Synapse 3D software (Fujifilm Medical Systems) followed by a manual inspection by a trained analyst. After inspection, minor manual measurements will be performed as required. The already used, validated, and free ImageJ software has been chosen for the final measurement and validation of the muscle surfaces [39] (Figure 2) on a digital imaging and communications in medicine format file as described in detail by Gomez-Perez et al [40].

The lumbar skeletal muscle index (LSMI) quantifies the overall muscle mass of the patient and is a recognized marker for sarcopenia [41]. It corresponds to the cross-sectional muscle area (cm^2^) at the L3 level that is used for the quantification of muscle mass, which is normalized by the squared height (m^2^) of the patient [33]. Thus, the LSMI is expressed in cm^2^/m^2^ and is positively correlated to the BMI. A second marker, skeletal muscle density, which quantifies the fat content, is based on the mean radio attenuation expressed in Hounsfield units (HUs) from a cross-sectional area at the L3 vertebral level, whose values are within the range of −29 to +150 HU [42,43]. These 2 markers are complementary, and it has been proposed to use their product to obtain the skeletal muscle gauge, which is expressed in HU cm^2^/m^2^ [44]. The skeletal muscle gauge correlates better with the outcomes in patients with some types of cancer [44-46].

The finger flexor muscle force is measured using a handgrip dynamometer (MAP 80K1; Instramed). During the testing procedure, the participants are seated with their tested elbow flexed to a right angle by their side, while the wrist is at a neutral position to minimize the involvement of peripheral muscles. Measurements of sarcopenia and muscle parameters will be conducted every 3 months during chemotherapy and every 6 months until 5 years during the follow-up.

In our study, sarcopenia will be diagnosed if patients present 2 of the 3 criteria as follows [26,47-49]: (1) an LSMI of <55.8 cm^2^/m^2^ for men or <38.9 cm^2^/m^2^ for women, (2) a maximum voluntary contraction of <32 kg for men or <22 kg for women, and (3) a short physical performances battery score of <8.

#### Cardiovascular and Respiratory Abnormalities

Clinical examinations will be conducted to identify signs of heart failure (dyspnea, edema of the lower limbs, crackles on pulmonary auscultation, weight gain, arterial hypertension, etc). Two additional tests will be performed: (1) LVEF which is the ratio (%) between the volume of ejected blood and the end diastolic volume of the left ventricle; this evaluation highlights the functioning of the left ventricle, which plays the role of maintaining an ejection volume adapted to peripheral blood requirements, and (2) pulmonary function tests, which measure respiratory volumes and flows by spirometry, flowmetry, plethysmography, and free diffusion of carbon monoxide; they can detect early abnormalities in lung capacity. Both evaluations will be conducted at the time point of inclusion, after 4 cycles of R-CHOP, and at the end of the treatment.

#### Frailty and Comorbidities

Frailty will be assessed by (1) the detection of comorbidities with the calculation of the Charlson score [48], (2) dependency using the G8 geriatric questionnaire [50], (3) cognitive disorders by calculating the mini mental status [51], and (4) autonomy through the Activities of Daily Living and the Instrumental Activities of Daily Living questionnaires [52,53]. These questionnaires will be offered only at the time point of inclusion.

#### Fatigue and Quality of Life

The overall quality of life will be assessed using the European Organisation for Research and Treatment of Cancer Quality of Life Questionnaire [54]. Depression will be assessed using the Geriatric Depression Scale questionnaire [55]. Fatigue will be assessed using the Multidimensional Fatigue Inventory questionnaire [56]. These questionnaires will be offered at the time of inclusion, after 4 cycles, at the end of the treatment, and then every 6 months until the end of the study.

### Physical Activity Program

**Figure 3:**
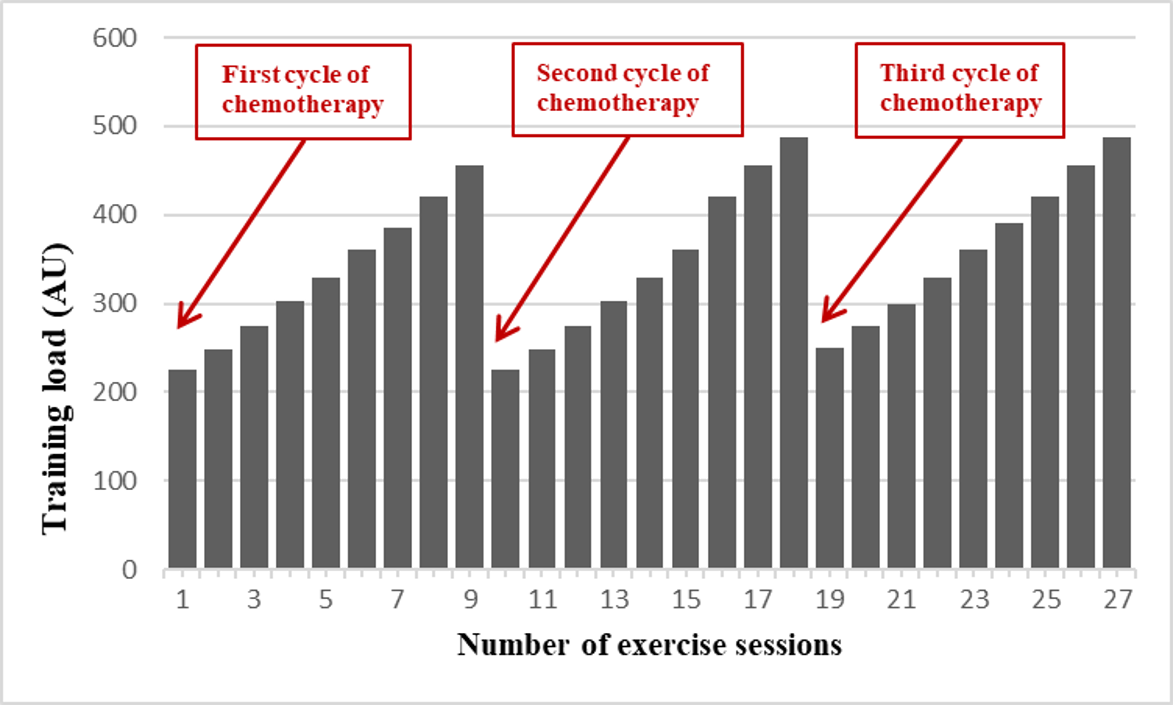
Representative sketch of the periodization to be followed for the exercise interventions (training load). AU: arbitrary unit.

The physical activity program is based on international recommendations in terms of its intensity and duration while also considering the side effects induced by chemotherapy [57]. A qualified physical activity teacher (eg, a kinesiologist or physical therapist) will be in charge of carrying out the sessions. To trigger the phenomenon of supercompensation [58-60], the workload of the exercise program will be modulated during the protocol. Thus, the exercise program will be characterized by a periodic and regular increase in workload during each cycle of chemotherapy planned by the physician (Figure 3).

The APA program can be described using the frequency, intensity, time, and type principle [61]. *Frequency*: participants will have to exercise 3 times a week. *Intensity*: to match the World Health Organization recommendations [62], the objective is to maintain an intensity of ≥6/10 (on Borg scale) as often as possible. *Time*: the APA program will last approximately 6 months, that is, the total duration of chemotherapy administration. *Type*: exercise should include resistance training (indoor with elastic bands, weight machines, and free weights) and endurance training, such as Nordic walking. According to the COVID-19 context, an amendment has been added to the protocol to offer the patient the possibility of following the same program through video calls with the supervision and direction of the APA teacher.

The training load (in arbitrary units) will be designed according to the Foster method, which is well known in the world of sports training and has the advantage of being able to quantify the training load in a simple way [63,64]. The principle is based on multiplying a patient’s feeling of the intensity of the effort (rating of perceived exertion; graded 0-10) by the duration of the session in minutes, thereby leading to the marker:

Training load AU = rating of perceived exertion 10 × duration min **(1)**

This approach to measure training load also provides a means to educate the patients to better assess their training load and to improve their independent practice. Another advantage of using this easy-to-use parameter of follow-up is that we can ensure an opportunity to design and perform the exercise program freely. Professionals and patients may choose the type of physical exercise and activity according to their preferences, equipment, and possibilities and still evaluate the training load as a parameter that can be used for interpatient comparisons.

### Statistical Analysis

A general description of the study population (demographics, disease history, and previous treatments) will be presented by group (experimental and control) and in total. For categorical variables, we will present the number of patients (n) and the percentage for each group in the study cohort. The significance of the statistical differences will be assessed using the chi-square test or Fisher exact test. For quantitative variables, we will report the median value or the mean value with the SD depending on the normality of the variable; the minima and maxima will also be indicated. Comparisons will be made by using the Fisher exact test, or Student test for small groups, or nonparametric Wilcoxon test, depending on the normality of the variables.

Survival curves will be plotted according to Kaplan-Meier estimates; median survival will be reported with their 95% CIs. The curves will be compared using the log rank test. The Cox model will be used to calculate the hazard ratios that will be reported with their 95% CIs. The completion rates of different Quality of Life Questionnaires (European Organization for the Research and Treatment of Cancer Quality of Life Questionnaire, Geriatric Depression Scale, Male Sexual Health questionnaire, and Brief Index of Sexual Functioning for Women) will be defined for each questionnaire and at each measurement point. The quality of life will be described for each evaluation (median value, mean value and SD, minima and maxima, and the frequency of the floor and ceiling effects) compared with the values obtained at the time of inclusion and then studied longitudinally using mixed-design analysis of variance for repeated measures. The degradation time will also be analyzed. The definitions that will be used are those proposed by Bonnetain et al [65]. Absolute prevalence—that is, the number of cases with an event—will be reported in addition to the prevalence (number of cases existing at the time of the assessment or number of patients observed at the same time). The incidence rate, which is the number of patients with a new event divided by the time spent by the patient during the follow-up period, will also be reported. Adherence, defined as the fraction of APA sessions that have been actually completed, will be reported. The cost analysis will be conducted over a 36-month period. The duration of our clinical follow-up is sufficient to allow us to assess the impact of APA on the care and quality of life of patients with DLBCL. Only the cost of hospitalization (medicines, surgery, or postcure rehabilitation) will be assessed. Data related to hospital stays will be made available through medical information departments of the participating centers. The cost will be estimated according to the rates provided by the Homogeneous Groups of Stays and will be considered as a daily supplement. The analyses will be performed using the SAS software (version 9.3, SAS Institute Inc), with a degree of significance set at 5%.

### Ethical Considerations

The study has been approved by the national research ethics committee (AU1636) and was registered as a clinical trial (NCT04670029) on December 16, 2020. This study will be conducted in accordance with the principles of the Declaration of Helsinki.

After the medical consultation, a research team member will provide patients with the study brochure and verbal description of the study and give them an opportunity to ask questions about the study. If the participant agrees, screening for eligibility to participate in the study is performed and signed informed consent will be obtained. The protocol complies with the SPIRIT (Standard Protocol Items: Recommendations for Interventional Trials) checklist. No compensation is included for participants within the framework of the study. All the care proposed is provided by the national social security. Travel to the study site is not financially covered for the participant.

## Results

We expect that APA will reduce relapse and complications in the short and medium term (infections, secondary cancers, and cardiovascular diseases induced by the treatments). This program is associated with nutritional and training-load monitoring and has the benefit of being reproducible from one center to another.

Recruitment, enrollment, and data collection began in February 2021, and 4 participants have been enrolled in the study as of July 2022. Data analysis will begin after the completion of data collection. Future outcomes will be published in peer-reviewed health-related research journals and presented at regional, national, congress, and state professional meetings. This publication is based on protocol version 1.1, August 3, 2020.

## Discussion

### Anticipated Principal Findings

The primary purpose of the PHARAOM study is to evaluate the effect of a standardized APA program applied concomitantly with R-CHOP chemotherapy on the survival of patients receiving first-line treatment for DLBCL.

The relationship between physical exercise and survival is poorly understood, especially in patients with DLBCL [27]. To the best of our knowledge, the PHARAOM study is the first randomized trial to propose a complete and repeated APA intervention in a population of patients receiving first-line treatment for DLBCL. The study findings should promote the growing body of evidence concerning the role of specific interventions such as APA in patients with cancers; for example, lymphoma.

Indeed, we expect an increase in the survival of patients who undertake an APA program initiated at diagnosis as reported in a previous study, where patients obtained benefit in survival only when completing physical activity before or after the first-line treatment [28,29]. We expect to obtain the benefit of APA in survival by implementing it at the beginning and during the first-line treatment period. The hitherto poorly studied and delicate character of the first-line treatment period explain why we experimented with an adjustable training-load process in our study. Our objective is to show that an APA program is conclusive for our population of patients with DLBCL, contrary to what is observed in the study conducted by Courneya et al [30] with this type of hematologic cancer.

Regarding the expected results of the evaluation, we anticipate that the results involved in the implementation of the intervention may be somewhat different, as the treatment period of the fragile population that will be assessed in this study may involve more complications previously cited, such as sarcopenia. Consequently, a lower survival rate in the sarcopenic group should be expected based on the studies reporting the outcomes in sarcopenic patients with solid tumors [66,67]. Similar results were obtained in patients with hematologic malignancies [20,27,68].

### Strengths and Limitations

We expect a major difficulty in adhering to the study owing to its innovative nature and the voluntary basis of the trial’s participation. In France, especially in the context of the COVID-19 pandemic, physicians are not yet involved in providing physical exercise–related care to their patients, mostly owing to a lack of information regarding this type of care. The same applies to patients themselves, where their spontaneous involvement in physical therapy during the treatment of their cancer is not common. Therefore, our study is faced with the challenge of helping to modify the management of a chronic illness (such as DLBCL) and at the same time, providing the tools to materialize national recommendations [69,70]. The COVID-19 pandemic has also disturbed the study development and has delayed the enrollment of patients owing to their immune frailty. Hematological conditions and human physical interactions through face-to-face exercise sessions are not compatible with the COVID-19 pandemic context; therefore, we propose the adoption of videoconference in the training sessions. The strength of this study is that it will be, to our knowledge, the first study to assess the prevalence of sarcopenia in both survival and clinical outcomes in this specific patient population. This is the first randomized trial to propose a complete and repetitive APA program for this frail population.

### Conclusions

Our study should provide new objective data that are needed for a deeper understanding of the effect of exercise on muscle mass and sarcopenia, which act as prognostic markers for patients with DLBCL. The frailty of the older adult population with DLBCL demands specific attention to the APA methodology used; therefore, we use a malleable training load in this study in an attempt to reduce (as far as possible) the chances of causing fatigue while inducing sufficient physical input in this fragile population.

The long-term objective of our research team is to develop and test APA interventions that improve survival and quality of life management in all cancer populations and can be implemented in various clinical settings. Findings from the PHARAOM study will consolidate refinement of APA interventions and trial design concerning physical activity in hematological cancer. Later inclusion of health economic modeling could shed further light on the long-term effects of intervention participation on the cost-effectiveness of this approach for APA promotion and integration in the national health system.

## Abbreviations

APA: adapted physical activity
CT: computed tomography
DLBCL: diffuse large B-cell lymphoma
EFS: event-free survival
HU: Hounsfield unit
LSMI: lumbar skeletal muscle index
LVEF: left ventricular ejection fraction
PFS: progression-free survival
PHARAOM: Physical Activity Program for the Survival of Elderly Patients with Lymphoma
R-CHOP: rituximab plus cyclophosphamide, hydroxydaunorubicin (doxorubicin), vincristine, and prednisone
SPIRIT: Standard Protocol Items: Recommendations for Interventional Trials

## Data Availability

The data sets generated and analyzed during this study are available from the corresponding author upon reasonable request.
